# Hospitalizations associated with influenza and respiratory syncytial virus among patients attending a network of private hospitals in South Africa, 2007–2012

**DOI:** 10.1186/s12879-014-0694-x

**Published:** 2014-12-16

**Authors:** Robert Kyeyagalire, Stefano Tempia, Adam L Cohen, Adrian D Smith, Johanna M McAnerney, Veerle Dermaux-Msimang, Cheryl Cohen

**Affiliations:** Nuffield Department of Population Health, University of Oxford, Oxford, UK; Influenza Division, Centers for Disease Control and Prevention, Atlanta, GA USA; Influenza Division, Centers for Disease Control and Prevention, Pretoria, South Africa; Center for Respiratory Diseases and Meningitis, National Institute for Communicable Diseases of the National Health Laboratory Service, Johannesburg, South Africa; School of Public Health, Faculty of Health Sciences, University of Witwatersrand, Johannesburg, South Africa

**Keywords:** Influenza virus, Respiratory syncytial virus, Hospitalization, South Africa

## Abstract

**Background:**

Influenza and respiratory syncytial virus (RSV) infection are common causes of lower respiratory tract illness. Data on their burden in low and middle-income settings and from Africa are scarce. We aimed to estimate age-specific rates of hospitalization attributable to influenza and RSV among patients attending private hospitals in South Africa during 2007–2012.

**Methods:**

We estimated annual age-specific rates of influenza- and RSV-associated hospitalization (that is respiratory hospitalizations likely due to influenza or RSV infection) by applying regression models to monthly administrative hospitalization data from a national private hospital group, using influenza and RSV surveillance data as covariates.

**Results:**

Estimated mean hospitalization rates associated with seasonal influenza were 75 (95% confidence interval (CI), 41–108) and 3 (95% CI, 2–5) per 100,000 person-years for all-respiratory and all-circulatory causes, respectively. Children <1 year and adults ≥75 years were the most affected, with influenza-associated all-respiratory hospitalization rates estimated at 255 (95% CI, 143–358) and 380 (95% CI, 227–506) per 100,000 person-years, respectively. Excess all-circulatory hospitalizations associated with seasonal influenza were only observed in adults ≥65 years. Annual hospitalization rates associated with RSV averaged an estimate of 223 (95% CI, 128–317) per 100,000 person-years for all-respiratory causes. Among children <1 year, RSV-associated all-respiratory hospitalization rate of 7,601 (95% CI, 4,312-10,817) per 100,000 person-years was estimated.

**Conclusions:**

Influenza and RSV substantially contributed to hospitalizations over the study period.

**Electronic supplementary material:**

The online version of this article (doi:10.1186/s12879-014-0694-x) contains supplementary material, which is available to authorized users.

## Background

In 2008, there were between 13 and 32 million cases of pneumonia attributed to influenza virus infection globally, with up to 110,000 deaths in children <5 years of age [1]. In the same age group, RSV infection accounted for 34 million hospitalizations and between 66,000 and 199,000 deaths [2]. The vast majority of these deaths likely due to influenza (influenza-associated) and RSV (RSV-associated) infection occurred in low and middle-income countries. The burden of influenza- and RSV-associated hospitalizations across different age-groups has been well documented in high-income countries [3]-[14]. However, such information are limited in low and middle-income settings.

The influenza season in South Africa is well-defined and occurs during the southern hemisphere winter months (May to August) [15], while peak activity of RSV is observed from February to May [16]. Recent estimates of hospitalization burden associated with influenza and RSV in South Africa are available only from one large population-based surveillance site [16],[17]. Since influenza and RSV infections are rarely confirmed by laboratory diagnosis we applied modeling approaches to estimate age-specific influenza- and RSV-associated hospitalizations among individuals hospitalized at a network of private hospitals in South Africa from 2007 through 2012.

## Methods

### Hospitalization data

We obtained anonymized hospitalization data on admission diagnoses, that were coded according to the *International Classification of Diseases, Tenth Revision* (ICD-10), from a large network of private hospitals covering seven of the nine Provinces in South Africa (Eastern Cape, Free State, Gauteng, KwaZulu-Natal, Mpumalanga, North West, and Western Cape), for the period 2007–2012. We used the ICD-10 codes to compile age-specific monthly hospitalization time-series for all-respiratory (ICD-10: J00-J99), pneumonia and influenza (P&I) (ICD-10: J10-J18 a subset of all-respiratory) and all-circulatory (ICD-10:I00-I99)) hospitalizations. Hospitalization data were analyzed in seven age categories: <1, 1–4, 5–19, 20–44, 45–64, 65–74 and ≥75 years of age.

### Population denominators

We obtained countrywide annual population estimates for each of the seven age-groups from Statistics South Africa for the years 2007–2012 [18]. We calculated the proportion of the population that had private health insurance by using available data on annual national health insurance coverage [18]. We then standardized it to the national population by age-group and health insurance coverage, based on the age-grouping data provided by the largest health insurance group in South Africa [19]. After obtaining the age-standardized number of individuals with private health insurance per year, we used annual market share data to estimate the proportion registered to seek care from the private hospital network under study. We estimated the private hospital group to have a market share of 30% among the insured population, by computing their reported annual number of hospital beds against the total number of private hospital beds in the country for each of the six years studied. The resulting market share was consistent with previous independent reports on private hospital market distribution in the country [20].

South Africa had an estimated population of 51.3 million in 2012; 16% of the population had private healthcare insurance, and the rest of the population sought care from public sector health facilities [18]. For this study we estimated the population served by the hospital group over the study period to represent 4.5 to 5.1 percent (1.9-2.5 million people) of the South Africa population.

### Influenza and RSV data

We acquired influenza and RSV virological data, for the period 2007–2012, from a national database (the National Health Laboratory Services (NHLS) corporate data warehouse) that includes all patients tested for respiratory viruses at any of the 268 networked public health laboratories in South Africa. Data on influenza type and subtype were not available from the national database; these data were obtained from the National Institute for Communicable Diseases (NICD)’s Viral Watch influenza surveillance programme, which receives samples from health practitioners across the country [15]. We considered an influenza type or subtype to be dominant during the influenza season when it accounted for more than 50% of the circulating viruses.

### Estimation of influenza- and RSV-associated hospitalizations

To estimate the influenza- (seasonal and pandemic) and RSV-associated hospitalizations, we fitted age-specific regression models with a Poisson distribution and an identity link to monthly all-respiratory, all-circulatory and P&I hospitalizations using methods previously described [21]. The identity link was selected because it is considered the most biologically plausible link to model the impact of pathogen circulation on mortality [22]-[25]. An identity link assumes additive (rather than multiplicative) effects of different pathogens on mortality. Through model selection procedures, we assessed the fit of models including higher order polynomials to represent more subtle time trends (1st to 6th degree) and additional harmonic terms representing annual and semi-annual periodicity (*sin(2t*_*i*_π*/12)* and *cos(2t*_*i*_π*/12)*; *sin(4t*_*i*_π*/12)* and *cos(4t*_*i*_π*/12)*). The final model was that for which the Akaike value was minimized, that is, the model that provided best fit to the data whilst maintaining parsimony. Variations of <5% on model estimates were observed while using a negative binomial model as compared to the Poisson model; nonetheless the latter provided a better fit to the data. We also considered b-splines (1 knot per month was best) instead of polynomial terms to model background seasonality but polynomial terms provided the best fit to the South African data, perhaps because of the relatively crude monthly resolution of the data.

The full model included covariates for time trends and seasonal variation as well as viral circulation as follows:

1 $$\begin{array}{l} E\left({Y_{i}}_{,t}\right)=\beta_{0,i}+\beta_{1,i}\left[t\right]+\beta_{2,i}\left[t^{2}\right]+\beta_{3,i}\left[t^{3}\right]+\beta_{4,i}\left[t^{4}\right]+\beta_{5,i}\left[\sin\left(2t\pi /12\right)\right]+\beta_{6,i}\left[\cos\left(2t\pi /12\right)\right]\\ +\beta_{7,i}\left[\mathrm{Seasonal}\_\mathrm{Influenza}\left(t\right)\right]+\beta_{8,i}\left[A\left(H1N1\right)pdm09\left(t\right)\right]+\beta_{9,i}\left[RSV\left(t\right)\right]+\varepsilon_{i,t} \end{array}$$

*E(Y*_*i,t*_*)* represents age-specific number of deaths in age group *i* and month *t*; β_*0,i*_ is the age-specific model constant; β_*1,i*_ to β_*4,i*_ are age-specific coefficients associated with time trends (linear to quartic polynomial terms); β_*5,i*_ and β_*6,i*_ are age-specific coefficients associated with harmonic terms accounting for annual background seasonal variations; β_*7,i*_ to β_*9,i*_ are age-specific coefficients representing the contribution of respiratory viruses to mortality (seasonal influenza (β_*7,i*_): including A(H1N1), A(H3N2) and B; pandemic influenza (β_*8,i*_): A(H1N1)pdm09; and RSV (β_*9,i*_)); and ε_*i,t*_ is the age-specific error term. *Seasonal_influenza(t)*, *A(H1N1)pdm09(t)* and *RSV(t)* are proxies for monthly viral activity, estimated as the monthly number of specimen testing positive for influenza or RSV over the annual number of specimens tested for the specific pathogen. We used standardization by the annual total of all specimens tested for the specific pathogen to reduce possible bias associated with differences in specimen sampling and laboratory methods over time [14],[21].

To estimate the excess hospitalization associated with influenza and RSV, we subtracted predicted monthly hospitalizations from a full model incorporating all viral terms from an expected baseline. The baseline was obtained by setting the viral covariates to zero and the annual excess hospitalizations were estimated as the sum of the monthly excess hospitalizations for each year. We obtained 95% confidence intervals (CI) for the estimated excess hospitalizations using bootstrap resampling on blocks of calendar years (12-month block resampling with replacement) over 1000 replications [21],[26]. For each resampled dataset we refitted the Poisson regression model and the 95% CI were obtained from the 2.5th and 97.5th percentiles of the estimated influenza- and RSV-associated hospitalizations from the 1000 resampled datasets. The statistical analysis was implemented using STATA version 12 (StataCorp, Texas, USA).

### Ethical considerations

Since the analysis used only de-identified and aggregated hospitalization and laboratory data, this study was considered to be exempt from human subjects’ ethics review.

## Results

### Rates of hospitalization

South Africa had a population of approximately 51.3 million people in 2012. Over the study period, an annual mean of 530,345 hospitalizations (range 491,155-576,634) across all age-groups occurred in the private hospital network for all causes, of which 51,899 (10%) were attributed to respiratory causes and 28,904 (6%) to circulatory causes. The mean annual rates of all-respiratory and P&I causes of hospitalization were highest in the <1 and ≥75 year age-groups and lowest in the 20–44 year age-group (Table 1). The rates of hospitalization attributed to circulatory illness increased with increasing age and were highest among those ≥75 years of age.

**Table 1:** **Mean annual hospitalization rates and population at risk among individuals with health insurance and served by the studied private hospital group in South Africa, 2007–2012**

Age-groups (in years)	Mean annual population in hospital group ^a^(range)	Cause of hospitalization		
		Respiratory ^b^(including pneumonia and influenza)	Pneumonia and influenza ^c^	Circulatory ^d^
		Mean annual rates ^e^(range ^f^)	Mean annual rates ^e^(range ^f^)	Mean annual rates ^e^(range ^f^)
<1	39529 (33009–43103)	29043 (25500–32697)	11542 (10148–13236)	226 (186–300)
1-4	162117 (132038–180020)	6083 (4741–7496)	1663 (1359–1846)	38 (30–46)
5-19	478789 (405825–519777)	2176 (1887–2564)	433 (337–519)	152 (139–187)
20-44	996137 (838835–1082658)	1322 (1172–1594)	409 (359–467)	841 (772–1036)
45-64	494431 (413592–534990)	2026 (1800–2293)	718 (623–781)	3795 (3511–4570)
65-74	103672 (79611–118082)	3742 (3434–4521)	1417 (1324–1718)	8677 (7546–10894)
≥75	48562 (26893–54317)	7507 (6595–9327)	3873 (3483–4773)	15175 (13990–18814)
All	2323237 (1929803–2532947)	2393 (2181–2767)	709 (654–778)	1938 (1795–2287)

^a^Determined by calculating proportion of age-specific population with health insurance, who are served by the hospital group under study, based on their market share estimates.

^b^Hospitalized with a diagnosis of International Classification of Diseases – Tenth Revision (ICD-10) codes of J00-J99.

^c^Hospitalized with a diagnosis of ICD-10: J10-J18.

^d^Hospitalized with a diagnosis of ICD-10: I00-I99.

^e^Hospitalization rate per 100,000 person-years.

^f^Rate ranges over the six years studied, 2007 to 2012.

### Influenza and RSV laboratory surveillance

**Figure 1:**
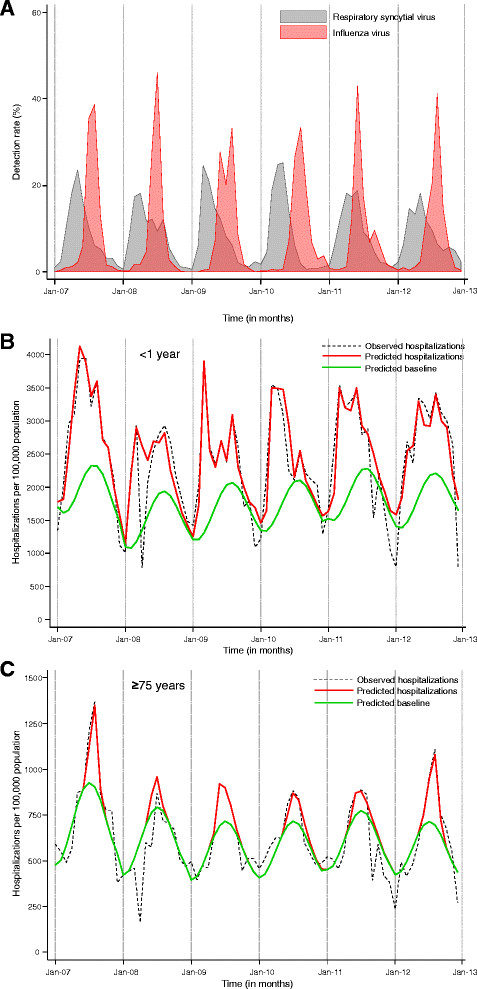
**Monthly detection of influenza and respiratory syncytial virus and hospitalization rates among individuals attending private hospitals in South Africa, 2007–2012. A**: Detection rate (i.e., monthly number of positive specimens divided by total specimens) of influenza and respiratory syncytial virus (all ages). **B**: Observed all-respiratory hospitalizations and predicted hospitalizations and baseline (Poisson model) by month in children <1 year of age. **C**: Observed all-respiratory hospitalizations and predicted hospitalizations and baseline (Poisson model) by month in individuals ≥75 years of age.

Means of 8,922 (range 4,720-15,321) and 6,640 (range 2,637-10,331) samples were tested annually for influenza and RSV, respectively. The mean annual number of specimens testing positive was 2,099 (23%) for influenza virus and 1,194 (18%) for RSV. During the study period, the influenza season peaked between June and August, while RSV peak activity was observed between February and April (Figure 1A). In 2009, a first wave of influenza peaked in June and was mainly due to A(H3N2), followed by a second influenza wave that peaked in August and was caused by A(H1N1)pdm09.

### Influenza- and RSV-associated hospitalizations

The estimated annual rates of all-respiratory hospitalizations attributed to seasonal influenza (excluding A(H1N1)pdm09) ranged from 54.2 to 111.9 per 100,000 person-years (Table 2). Annual circulatory hospitalizations associated with influenza were estimated to range from 2.3 to 4.8 per 100,000 person-years. Rates of RSV-associated hospitalizations among the <20 year age-group ranged from 195.9 to 262.7 per 100,000 person-years for all-respiratory illnesses over the study period.

**Table 2:** **Annual variation in influenza types/subtypes and estimated annual influenza- and respiratory syncytial virus-associated hospitalizations among individuals (all ages) attending a private hospital group in South Africa, 2007–2012**

Year	Estimated hospitalizations				
	Influenza (excluding A(H1N1)pdm09 in 2009)			RSV	
	Dominant type/subtype	Number ^a^	Rate ^b^	Number ^a^	Rate ^b^
**All-respiratory (including pneumonia and influenza)**					
2007	A (H3N2)	2151	112	5098	263
2008	A (H1N1)	1707	73	4889	207
2009	A (H3N2)	1934	79	6432	261
2010	B	1660	66	4987	197
2011	A (H1N1)pdm09	1637	72	4475	196
2012	A (H3N2)	1282	54	5112	216
**Pneumonia and influenza only**					
2007	A (H3N2)	1136	57	2011	102
2008	A (H1N1)	902	37	1929	80
2009	A (H3N2)	1022	41	2538	101
2010	B	877	34	1968	76
2011	A (H1N1)pdm09	865	37	1766	75
2012	A (H3N2)	677	28	2017	83
**All-circulatory**					
2007	A (H3N2)	92	5	…	…
2008	A (H1N1)	73	3	…	…
2009	A (H3N2)	83	3	…	…
2010	B	71	3	…	…
2011	A (H1N1)pdm09	70	3	…	…
2012	A (H3N2)	55	2	…	…

^a^Estimated number of patients served in the private hospital group per cause of hospitalization per year.

^b^Hospitalization rate per 100,000 person-years.

Estimated mean annual all-respiratory hospitalization rates associated with seasonal influenza were highest among children aged <1 year (255 per 100,000 person-years) and individuals aged ≥75 years (380 per 100 000 person years) (Table 3). Seasonal influenza-associated all-circulatory hospitalizations were only estimated among individuals ≥65 years of age.

**Table 3:** **Estimated mean annual influenza- and respiratory syncytial virus-associated hospitalizations by age group among individuals attending a private hospital group in South Africa, 2007–2012**

Age groups (in years)	Influenza (mean)						Respiratory syncytial virus (Mean)		
	Seasonal			A(H1N1)pdm09 in 2009					
	Number(95% CI)	Rate ^a^(95% CI)	Percentage over total hospitalizations(95% CI)	Number(95% CI)	Rate ^a^(95% CI)	Percentage over total hospitalizations(95% CI)	Number(95% CI)	Rate ^a^(95% CI)	Percentage over total hospitalizations(95% CI)
**All-respiratory**									
<1	99 (56–142)	255 (143–358)	1.2 (0.8-1.6)	260 (148–372)	621 (354–888)	3.1 (2.2-4.0)	2990 (1704–4276)	7601 (4312–10817)	36.2 (25.3-47.1)
1-4	326 (196–456)	205 (121–282)	4.3 (3.0-5.6)	197 (118–276)	116 (70–162)	2.4 (1.7-3.1)	1902 (1141–2663)	1182 (704–1643)	25.7 (17.7-33.7)
5-19	261 (117–405)	55 (25–84)	2.7 (1.9-3.5)	1183 (532–1834)	233 (105–361)	11.8 (8.1-15.5)	272 (122–422)	57 (26–88)	2.8 (1.9-3.7)
20-44	366 (179–553)	37 (18–55)	2.9 (2.0-3.8)	922 (452–2392)	87 (43–131)	7.0 (4.8-9.2)	…	…	…
45-64	341 (198–484)	70 (40–98)	3.5 (2.3-4.7)	588 (341–835)	112 (65–159)	5.9 (3.8-8.0)	…	…	…
65-74	154 (92–216)	154 (89–208)	4.1 (2.6-5.6)	148 (89–207)	139 (73–163)	4.0 (2.5-5.5)	…	…	…
75+	178 (110–246)	380 (227–506)	5.2 (3.5-6.9)	61 (38–84)	117 (73–163)	1.7 (1.1-2.3)	…	…	…
All	1725 (949–2501)	75 (41–108)	3.1 (2.2-4.0)	3359 (1718–5001)	136 (70–203)	5.9 (4.1-7.7)	5164 (2968–7360)	223 (128–317)	9.3 (6.4-12.2)
**Pneumonia and influenza**									
<1	94 (54–134)	241 (136–340)	6.9 (4.8-9.0)	204 (116–292)	488 (278–697)	12.2 (8.5-15.9)	1201 (685–1717)	3055 (1732–4345)	34.6 (24.2-45.0)
1-4	125 (75–175)	78 (46–108)	8.1 (5.6-10.6)	187 (112–262)	110 (66–154)	13.5 (9.3-17.7)	735 (441–1029)	457 (272–635)	25.9 (17.9-33.9)
5-19	129 (58–200)	27 (12–42)	7.6 (5.2-10.0)	678 (305–1051)	133 (60–207)	38.9 (26.8-51.0)	100 (45–155)	21 (9–32)	5.9 (4.1-7.7)
20-44	198 (97–299)	20 (10–30)	7.2 (4.9-9.5)	481 (236–726)	45 (22–69)	12.0 (8.2-15.8)	…	…	…
45-64	195 (113–277)	40 (23–56)	7.8 (5.1-10.5)	278 (161–395)	53 (31–75)	8.1 (5.3-10.9)	…	…	…
65-74	85 (51–119)	85 (49–115)	6.1 (3.8-8.4)	83 (50–116)	78 (47–110)	5.9 (3.7-8.1)	…	…	…
75+	120 (74–166)	256 (153–341)	6.9 (4.6-9.2)	29 (18–40)	56 (35–77)	2.1 (1.4-2.8)	…	…	…
All	946 (522–1370)	41 (22–59)	7.7 (5.4-10.0)	1940 (998–2882)	77 (41–117)	11.3 (7.9-14.7)	2036 (1171–2901)	86 (50–125)	12.2 (8.4-16.0)
**All-circulatory**									
<1	…	…	…	…	…	…	…	…	…
1-4	…	…	…	…	…	…	…	…	…
5-19	…	…	…	…	…	…	…	…	…
20-44	…	…	…	…	…	…	…	…	…
45-64	…	…	…	…	…	…	…	…	…
65-74	23 (10–36)	23 (10–34)	0.2 (0.1-0.3)	91 (55–127)	86 (52–120)	1.0 (0.6-1.4)	…	…	…
75+	50 (25–75)	107 (51–154)	0.7 (0.5-0.9)	14 (9–19)	28 (17–37)	0.2 (0.1-0.3)	…	…	…
All	73 (35–111)	3 (2–5)	0.1 (0.06-0.14)	105 (63–147)	4 (3–6)	0.1 (0.5-0.15)	…	…	…

^a^Hospitalization rate per 100,000 person-years.

In 2009 the estimated rate of all-respiratory hospitalizations associated with influenza A(H1N1)pdm09 was 136 per 100,000 person-years, almost two times higher than the mean influenza-associated hospitalizations during non-pandemic years (Table 3). The rates of hospitalizations associated with influenza A(H1N1)pdm09 among individuals >5 years of age were higher than mean seasonal influenza hospitalization rates (especially in the 5–19 age group), but lower among elderly individuals.

The estimated mean annual rates of RSV-associated all-respiratory hospitalizations decreased with age; and it was highest among <1 year age-group (7,601 per 100,000 person-years) and lowest among 5–19 year age-group (57 per 100,000 person-years) (Table 3). Similar trends were observed among pneumonia and influenza cases that were associated with RSV. Our model did not estimate any RSV-associated hospitalizations among individuals ≥20 years of age.

## Discussion

Influenza and RSV substantially contributed to hospitalizations in South Africa throughout the study period. Hospitalization rates associated with seasonal influenza were highest among children <1 year of age and individuals aged ≥75 years, while RSV mostly affected children <5 years of age. We did not estimate any RSV-associated hospitalizations among individuals aged ≥20 years.

Elevated influenza-associated hospitalizations have been reported among infants and elderly individuals in other studies conducted in the United States and Europe [8],[13],[14]. Estimated rates of influenza-associated hospitalizations per 100,000 person-years in South Africa among children <1 (255) and 1–4 (205) years of age for all-respiratory causes were elevated, compared with the United States (151 in children <1 year of age and 39 among children 1–4 years of age for respiratory and circulatory cause of hospitalization) [14]. Estimated rates of influenza-associated P&I hospitalizations (41 per 100,000 person-year) observed in our study were similar to estimates of influenza-associated hospitalizations (49 and 54 per 100,000 person-year in 2010 and 2011, respectively) derived from a large population-based public hospital surveillance site in Soweto, South Africa, where patients presenting with acute lower respiratory tract infection were systematically enrolled and tested for influenza from 2009 through 2011 [17].

Rates of influenza A(H1N1)pdm09-associated all-respiratory hospitalizations in 2009 in South Africa were estimated to be approximately double those observed during non-pandemic years and disproportionately affected young individuals (especially the 5–19 year age-group), with the elderly being the least affected. Studies on the 2009 influenza pandemic previously conducted in the United States [27],[28], Canada [29], Argentina [30] and Italy [31] observed similar patterns.

We identified an elevated burden of RSV compared to influenza infection among children <5 years of age. A study conducted in children aged <5 years with respiratory infections that attended public sector hospitals in South Africa showed a 30% positivity proportion for RSV compared to only 4% for influenza [32]. Another study conducted in the United States using a methodology similar to ours found RSV-associated hospitalizations to be 16 times higher than influenza among children <1 year of age [14],[33]. In our study, estimated mean annual rates per 100,000 person-years of RSV-associated P&I hospitalizations among children <1 (3,055) and 1–4 (457) years of age were similar to those observed from a population-based surveillance site in Soweto, South Africa, in 2010–2011 (2,400-3,200 in children <1 year of age and 500–600 in children aged 1–4 years) [16].

Our model did not estimate any excess hospitalizations associated with RSV among elderly individuals, although other studies have previously reported morbidity and mortality among adults infected with laboratory-confirmed RSV [34],[35]. Studies conducted in Kenya and South Africa that compared the RSV prevalence among patients hospitalized with severe acute respiratory illness (SARI) to controls found that RSV infection was associated with hospitalization among children <5 years of age, but no association was found among individuals ≥5 years old [36],[37]. Studies conducted in Egypt, Guatemala, Kenya and Thailand, where patients of all ages hospitalized with acute lower-respiratory tract infections were systematically enrolled and tested using molecular techniques, reported RSV detection rates of <1-5% among individuals ≥50 or ≥65 years of age compared to RSV detection rates of >20% in infants and young children [38]-[40]. In South Africa in 2009–2010, the RSV detection rate among patients hospitalized with SARI decreased from 26.8% among infants <1 year of age to 0.9% among individuals ≥65 years, compared with an influenza detection rate of approximately 8-12% across all age groups in the same study population [33]. This may suggest that although RSV can be detected among older children and adults, it may play a less important role as a pathogen in this group.

A recent study conducted to estimate adult mortality associated with influenza and RSV in South Africa (using a methodology similar to ours) found only influenza- and not RSV-associated mortality among individuals older than 44 years of age [41]. While cases of RSV-associated hospitalization and mortality among elderly individuals are expected to occur in South Africa, our modelling approach may have failed to statistically estimate a small number of cases. Other national estimates of influenza- and RSV-associated mortality among adult and elderly individuals have been determined mainly using modelling approaches similar to ours and in settings where the influenza and RSV seasons are more synchronous [7],[42]. Ecological studies conducted in settings similar to ours, where influenza and RSV peak activities are not synchronous, may assist in better differentiating the relative burden of these pathogens especially in adults. While in our study we report low to moderate RSV-associated hospitalization among individuals ≥5 years of age, clinical diagnosis and surveillance for both influenza and RSV should be continued and strengthened, and may be leveraged to better understand the burden and severity associated with RSV infection in older age groups.

Our study has limitations that warrant discussion. Firstly, the lack of weekly hospitalization data may have hindered the ability to more accurately estimate the relative contribution of RSV and influenza virus on hospitalizations. In addition, the monthly temporal resolution of our data did not allow us to include viral circulation proxies for each season. Our approach may underestimate the annual variation of influenza-associated hospitalizations in our study. Secondly, lack of availability of influenza and RSV incidence data hampered our ability to consider more refined indicators of respiratory virus activity in our time series models as reported by Goldstein et al. [25],[26]. Thirdly, we were not powered to estimate the association of influenza and RSV circulation with more refined causes of hospitalization. In addition, data on the circulation of respiratory viruses other than influenza and RSV were not available for the entire study period and were not included in our study. This may potentially overestimate the hospitalizations attributed to influenza or RSV infection. Nonetheless, the estimates obtained in our study are similar to those obtained from case-based studies in South Africa [16],[17]. Furthermore case-based surveillance implemented among patients hospitalized with severe acute respiratory illness (SARI) in South Africa from 2009 indicated peak activities of human metapneumovirus and parainfluenza virus type 3 after the influenza season (September to November) with an annual detection rate among SARI cases of approximately 4% for each pathogen. Given the different seasonality of these pathogens minimal effect is expected on the influenza- and RSV-associated hospitalizations estimated with our model. All other respiratory viruses tested under the SARI surveillance programme (e.g. adenovirus, enterovirus, parainfluenza virus types 1 and 2, and rhinovirus) were detected year-around without clear seasonality [33]. Fourthly, we could only estimate influenza- and RSV-associated hospitalization using data from a proportion of private hospitals in South Africa as national hospitalization data, from all private and public health facilities, were not available. In addition we estimated the service population of the hospital group used in this study based on available data on number of individuals insured and market share of this hospital group. The representativeness of our estimations for national population is therefore uncertain. Nonetheless, our incidence rate estimates were similar to those obtained using active, prospective surveillance from a large population-based public sentinel hospital [16],[17], and may be reasonably representative of the total population.

## Conclusion

Our study found that influenza and RSV were associated with substantial hospitalization rates in South Africa, with influenza responsible for hospitalizations across all age-groups and RSV affecting mainly individuals <5 years of age. Vaccination remains the most effective method of preventing influenza virus infection [43], and the effectiveness of RSV candidate vaccines is being evaluated [44],[45] and when available could potentially substantially reduce the elevated burden of hospitalization especially in young children.

## Authors’ original submitted files for images

Below are the links to the authors’ original submitted files for images. Authors’ original file for figure 1 Authors’ original file for figure 2

## Abbreviations

NICD: National Institute of Communicable Diseases, of South Africa
P&I: Pneumonia and influenza
RSV: Respiratory syncytial virus
SARI: Severe acute respiratory illness
