# Supplementary figures and images for: Hospitalizations associated with influenza and respiratory syncytial virus among patients attending a network of private hospitals in South Africa, 2007–2012
Source: BMC Infect Dis. 2014 Dec 16;14:694. doi: 10.1186/s12879-014-0694-x (PMC4278267; doi:10.1186/s12879-014-0694-x)

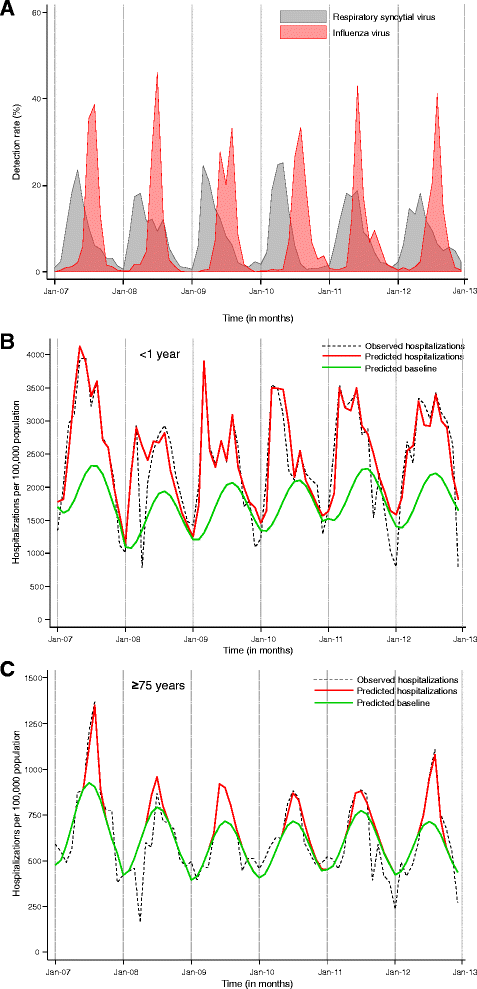

Supplement: Supplementary file 1 — Authors’ original file for figure 1 [file 12879_2014_694_MOESM1_ESM.gif]
