# Supplementary material for: Hospitalizations associated with influenza and respiratory syncytial virus among patients attending a network of private hospitals in South Africa, 2007–2012
Source: BMC Infect Dis. 2014 Dec 16;14:694. doi: 10.1186/s12879-014-0694-x (PMC4278267; doi:10.1186/s12879-014-0694-x)
Supplement: Supplementary file 2 — Authors’ original file for figure 2 [file 12879_2014_694_MOESM2_ESM.docx]

Table 1: Mean annual hospitalization rates and population at risk among individuals with health insurance and served by the studied private hospital group in South Africa, 2007-2012.

| **Age-groups (in years)** | **Mean annual population in hospital group^a^ (range)** | **Cause of hospitalization** | | |
| --- | --- | --- | --- | --- |
|  |  | Respiratory^b^ (including pneumonia and influenza) | Pneumonia and Influenza^c^ | Circulatory^d^ |
|  |  | Mean annual rates^e^ (range^f^) | Mean annual rates^e^ (range^f^) | Mean annual rates^e^ (range^f^) |
| <1 | 39529 (33009-43103) | 29043 (25500-32697) | 11542 (10148-13236) | 226 (186-300) |
| 1-4 | 162117 (132038-180020) | 6083 (4741-7496) | 1663 (1359-1846) | 38 (30-46) |
| 5-19 | 478789 (405825-519777) | 2176 (1887-2564) | 433 (337-519) | 152 (139-187) |
| 20-44 | 996137 (838835-1082658) | 1322 (1172-1594) | 409 (359-467) | 841 (772-1036) |
| 45-64 | 494431 (413592-534990) | 2026 (1800-2293) | 718 (623-781) | 3795 (3511-4570) |
| 65-74 | 103672 (79611-118082) | 3742 (3434-4521) | 1417 (1324-1718) | 8677 (7546-10894) |
| ≥75 | 48562 (26893-54317) | 7507 (6595-9327) | 3873 (3483-4773) | 15175 (13990-18814) |
| All | 2323237 (1929803-2532947) | 2393 (2181-2767) | 709 (654-778) | 1938 (1795-2287) |

^a^ Determined by calculating proportion of age-specific population with health insurance, who are served by the hospital group under study, based on their market share estimates.

^b^ Hospitalized with a diagnosis of International Classification of Diseases – Tenth Revision (ICD-10) codes of J00-J99

^c^ Hospitalized with a diagnosis of ICD-10: J10-J18

^d^ Hospitalized with a diagnosis of ICD-10: I00-I99

^e^ Hospitalization rate per 100,000 person-years

^f^ Rate ranges over the six years studied, 2007 to 2012

**Table 2:** Annual variation in influenza types/subtypes and estimated annual influenza- and respiratory syncytial virus-associated hospitalizations among individuals (all ages) attending a private hospital group in South Africa, 2007-2012.

| Year | Estimated hospitalizations | | | | |
| --- | --- | --- | --- | --- | --- |
|  | Influenza (excluding A(H1N1)pdm09 in 2009) | | | RSV | |
|  | Dominant type/subtype | Number^a^ | Rate^b^ | Number^a^ | Rate^b^ |
| All-respiratory (including pneumonia and influenza) | | | | | |
| 2007 | A (H3N2) | 2151 | 112 | 5098 | 263 |
| 2008 | A (H1N1) | 1707 | 73 | 4889 | 207 |
| 2009 | A (H3N2) | 1934 | 79 | 6432 | 261 |
| 2010 | B | 1660 | 66 | 4987 | 197 |
| 2011 | A (H1N1)pdm09 | 1637 | 72 | 4475 | 196 |
| 2012 | A (H3N2) | 1282 | 54 | 5112 | 216 |
| Pneumonia and influenza only | | | | | |
| 2007 | A (H3N2) | 1136 | 57 | 2011 | 102 |
| 2008 | A (H1N1) | 902 | 37 | 1929 | 80 |
| 2009 | A (H3N2) | 1022 | 41 | 2538 | 101 |
| 2010 | B | 877 | 34 | 1968 | 76 |
| 2011 | A (H1N1)pdm09 | 865 | 37 | 1766 | 75 |
| 2012 | A (H3N2) | 677 | 28 | 2017 | 83 |
| All-circulatory | | | | | |
| 2007 | A (H3N2) | 92 | 5 | … | … |
| 2008 | A (H1N1) | 73 | 3 | … | … |
| 2009 | A (H3N2) | 83 | 3 | … | … |
| 2010 | B | 71 | 3 | … | … |
| 2011 | A (H1N1)pdm09 | 70 | 3 | … | … |
| 2012 | A (H3N2) | 55 | 2 | … | … |

^a^ Estimated number of patients served in the private hospital group per cause of hospitalization per year.

^b^ Hospitalization rate per 100,000 person-years.

**Table 3:** Estimated mean annual influenza- and respiratory syncytial virus-associated hospitalizations by age group among individuals attending a private hospital group in South Africa, 2007-2012.

| Age groups (in years) | Influenza (mean) | | | | | | Respiratory syncytial virus (Mean) | | |
| --- | --- | --- | --- | --- | --- | --- | --- | --- | --- |
|  | Seasonal | | | A(H1N1)pdm09 in 2009 | | |  |  |  |
|  | Number  (95% CI) | Rate^a^  (95% CI) | Percentage over total hospitalizations  (95% CI) | Number  (95% CI) | Rate^a^  (95% CI) | Percentage over total hospitalizations  (95% CI) | Number  (95% CI) | Rate^a^  (95% CI) | Percentage over total hospitalizations  (95% CI) |
| All-respiratory | | | | | | | | | |
| <1 | 99 (56-142) | 255 (143-358) | 1.2 (0.8-1.6) | 260 (148-372) | 621 (354-888) | 3.1 (2.2-4.0) | 2990 (1704-4276) | 7601 (4312-10817) | 36.2 (25.3-47.1) |
| 1-4 | 326 (196-456) | 205 (121-282) | 4.3 (3.0-5.6) | 197 (118-276) | 116 (70-162) | 2.4 (1.7-3.1) | 1902 (1141-2663) | 1182 (704-1643) | 25.7 (17.7-33.7) |
| 5-19 | 261 (117-405) | 55 (25-84) | 2.7 (1.9-3.5) | 1183 (532-1834) | 233 (105-361) | 11.8 (8.1-15.5) | 272 (122-422) | 57 (26-88) | 2.8 (1.9-3.7) |
| 20-44 | 366 (179-553) | 37 (18-55) | 2.9 (2.0-3.8) | 922 (452-2392) | 87 (43-131) | 7.0 (4.8-9.2) | … | … | … |
| 45-64 | 341 (198-484) | 70 (40-98) | 3.5 (2.3-4.7) | 588 (341-835) | 112 (65-159) | 5.9 (3.8-8.0) | … | … | … |
| 65-74 | 154 (92-216) | 154 (89-208) | 4.1 (2.6-5.6) | 148 (89-207) | 139 (73-163) | 4.0 (2.5-5.5) | … | … | … |
| 75+ | 178 (110-246) | 380 (227-506) | 5.2 (3.5-6.9) | 61 (38-84) | 117 (73-163) | 1.7 (1.1-2.3) | … | … | … |
| All | 1725 (949-2501) | 75 (41-108) | 3.1 (2.2-4.0) | 3359 (1718-5001) | 136 (70-203) | 5.9 (4.1-7.7) | 5164 (2968-7360) | 223 (128-317) | 9.3 (6.4-12.2) |
| Pneumonia and influenza | | | | | | | | | |
| <1 | 94 (54-134) | 241 (136-340) | 6.9 (4.8-9.0) | 204 (116-292) | 488 (278-697) | 12.2 (8.5-15.9) | 1201 (685-1717) | 3055 (1732-4345) | 34.6 (24.2-45.0) |
| 1-4 | 125 (75-175) | 78 (46-108) | 8.1 (5.6-10.6) | 187 (112-262) | 110 (66-154) | 13.5 (9.3-17.7) | 735 (441-1029) | 457 (272-635) | 25.9 (17.9-33.9) |
| 5-19 | 129 (58-200) | 27 (12-42) | 7.6 (5.2-10.0) | 678 (305-1051) | 133 (60-207) | 38.9 (26.8-51.0) | 100 (45-155) | 21 (9-32) | 5.9 (4.1-7.7) |
| 20-44 | 198 (97-299) | 20 (10-30) | 7.2 (4.9-9.5) | 481 (236-726) | 45 (22-69) | 12.0 (8.2-15.8) | … | … | … |
| 45-64 | 195 (113-277) | 40 (23-56) | 7.8 (5.1-10.5) | 278 (161-395) | 53 (31-75) | 8.1 (5.3-10.9) | … | … | … |
| 65-74 | 85 (51-119) | 85 (49-115) | 6.1 (3.8-8.4) | 83 (50-116) | 78 (47-110) | 5.9 (3.7-8.1) | … | … | … |
| 75+ | 120 (74-166) | 256 (153-341) | 6.9 (4.6-9.2) | 29 (18-40) | 56 (35-77) | 2.1 (1.4-2.8) | … | … | … |
| All | 946 (522-1370) | 41 (22-59) | 7.7 (5.4-10.0) | 1940 (998-2882) | 77 (41-117) | 11.3 (7.9-14.7) | 2036 (1171-2901) | 86 (50-125) | 12.2 (8.4-16.0) |
| All-circulatory | | | | | | | | | |
| <1 | … | … | … | … | … | … | … | … | … |
| 1-4 | … | … | … | … | … | … | … | … | … |
| 5-19 | … | … | … | … | … | … | … | … | … |
| 20-44 | … | … | … | … | … | … | … | … | … |
| 45-64 | … | … | … | … | … | … | … | … | … |
| 65-74 | 23 (10-36) | 23 (10-34) | 0.2 (0.1-0.3) | 91 (55-127) | 86 (52-120) | 1.0 (0.6-1.4) | … | … | … |
| 75+ | 50 (25-75) | 107 (51-154) | 0.7 (0.5-0.9) | 14 (9-19) | 28 (17-37) | 0.2 (0.1-0.3) | … | … | … |
| All | 73 (35-111) | 3 (2-5) | 0.1 (0.06-0.14) | 105 (63-147) | 4 (3-6) | 0.1 (0.5-0.15) | … | … | … |

^a^Hospitalization rate per 100,000 person-years.
